# Utilizing whole genome sequencing data for machine learning driven prediction of antibiotic resistance in *Escherichia coli*

**DOI:** 10.3389/fmicb.2026.1842717

**Published:** 2026-04-28

**Authors:** Fang Wan, Wanning Tong, Weiwei Wu, Jia Liu, Hao Huang, Pei Zhang, Wenqing Jiang

**Affiliations:** 1 Department of Laboratory Medicine, Nanjing First Hospital, Nanjing Medical University, Nanjing, Jiangsu, China; 2 Department of Respiratory and Critical Care Medicine, PLA Naval Medical Center, Shanghai, China; 3 Dinfectome Inc., Nanjing, Jiangsu, China; 4 Department of Neurosurgery, Wujin Hospital Affiliated with Jiangsu University, Changzhou, Jiangsu, China

**Keywords:** antimicrobial resistance, *Escherichia coli*, feature importance, machine learning, whole-genome sequencing

## Abstract

**Background:**

Antimicrobial resistance (AMR) is an escalating global public health threat that substantially undermines the effectiveness of standard anti-infective therapies and increases the risk of adverse clinical outcomes.

**Methods:**

We analyzed whole-genome sequencing (WGS) data from 1952 *Escherichia coli* isolates with AST phenotypes. Gene, single-nucleotide polymorphisms (SNPs), and k-mer features were extracted to train machine-learning classifiers for predicting resistance to 10 common antibiotics. Model performance was evaluated using the area under the receiver operating characteristic curve (AUC) and other classification metrics.

**Results:**

Across all antibiotics and feature representations, model AUCs ranged from 0.6691 to 0.9879. Gene-based and integrated-feature models showed superior and stable performance (AUC 0.8936–0.9787 and 0.8888–0.9879, respectively) compared with SNP-based models and k-mer models. Prediction performance was near-saturated for aminoglycosides (GEN and TOB) and ciprofloxacin (CIP), whereas β-lactams exhibited greater heterogeneity, with amoxicillin/clavulanate (AMX/CLA) exhibiting the lowest AUC. Feature-importance analysis highlighted 40 core genes that were highly concordant with established resistance mechanisms, including *aac(3)-IIg*/*aac(3)-IId* for aminoglycosides, *blaTEM*, *blaSHV-12*, *blaCTX-M-14*, and *blaOXA* for β-lactams, and *tetR(A)*/*tet(A)* for tetracyclines.

**Conclusion:**

This study demonstrated that machine learning models built on WGS data can accurately and efficiently predict resistance phenotypes to 10 commonly used antibiotics in *Escherichia coli*. Among the evaluated feature representations, gene-based and integrated multi-feature approaches yielded the most robust and reliable performance across antibiotic-specific tasks, highlighting the practical utility of WGS-derived genomic features for rapid AMR phenotype prediction and future clinical decision-support applications.

## Introduction

In recent years, the continuous rise of antimicrobial resistance (AMR) has been compromising the effectiveness of antimicrobial therapies, resulting in poorer clinical outcomes and imposing a greater economic burden on healthcare systems (Sakagianni et al., 2023). Global assessments of the burden of AMR indicate that in 2019 alone, approximately 4.95 million deaths were associated with AMR, with 1.27 million directly attributable to bacterial AMR (Zhang et al., 2024). Among clinically significant pathogens, *Escherichia coli*, one of the most common Gram-negative bacteria, accounts for a substantial proportion of both community-acquired and hospital-acquired infections, including urinary tract infections, intra-abdominal infections, and bloodstream infections (Bai et al., 2020; Park et al., 2022; Doua et al., 2024). Furthermore, the widespread resistance of *Escherichia coli* to commonly used antibiotics, including β-lactams, fluoroquinolones, aminoglycosides, and folate pathway inhibitors, has increased the risk of empirical treatment failures. This underscores the urgent need for rapid, scalable molecular diagnostics for resistance and tools to support precise therapeutic decision-making.

With the rapid advances in sequencing technologies, researchers have gained an increasingly refined understanding of the relationship between genotypes and AMR phenotypes. Machine learning (ML) approaches based on whole-genome sequencing (WGS) offer a scalable and potentially more accurate framework for rapid AMR prediction (Kim et al., 2022). Previous studies have demonstrated that WGS derived features can be leveraged to achieve reasonably accurate resistance prediction across a range of bacterial species (Van Camp et al., 2020; Liu et al., 2025). However, early AMR phenotype prediction models predominantly used known antibiotic resistance genes (ARGs) as input features, providing relatively rapid and interpretable inference of resistance (Renz et al., 2025). Nevertheless, approaches that rely solely on known ARGs (or are restricted to established resistance mechanisms) are prone to missed detections and misclassification due to incomplete reference databases and inter-strain variability (Bialobzyski, 2025).

Mutation-level features such as single-nucleotide polymorphisms (SNPs) can capture resistance determinants that are not well explained by gene presence/absence alone, including alterations in drug targets and regulatory pathways, thereby potentially reducing false negatives and improving prediction (Shahreen et al., 2025). K mer-based representations require no predefined genes or loci and can learn phenotype-associated patterns across the genome, but they are typically high-dimensional and less interpretable, and mapping predictive k-mer signatures to specific genetic factors is often non-trivial (Yang et al., 2025). Therefore, a systematic comparison of gene, SNP, and k-mer feature sets across antibiotic-specific resistance prediction tasks in *E. coli* is important for developing AMR models that balance accuracy and interpretability.

## Materials and methods

### Strain WGS data and AST phenotype data acquisition

Antibiotic resistance phenotypes for *Escherichia coli* and their corresponding assembly accession numbers were acquired from the BV-BRC database, yielding phenotype records for 15 antibiotics. Using these assembly accession identifiers, the associated raw whole-genome sequencing data were downloaded from the NCBI database. Antibiotics represented by fewer than 100 susceptible isolates were excluded from downstream analyses. After filtering, a benchmark dataset comprising 1952 *E. coli* genomes with matched susceptibility phenotypes was constructed for model development, covering the 10 antibiotics listed in Table 1.

**TABLE 1:** Benchmark dataset of AMR phenotypes in *Escherichia coli.*

Antibiotic	Abbreviation	Susceptible	Resistant
Cefepime	FEP	1,201	211
Tobramycin	TOB	1,214	368
Cefoxitin	FOX	765	402
Ceftriaxone	CRO	696	444
Gentamicin	GEN	2,765	580
Tetracycline	TET	440	599
Ceftazidime	CAZ	2,349	713
Ciprofloxacin	CIP	2,195	1,034
Amoxicillin/clavulanic acid	AMX/CLA	2,070	1,215
Ampicillin	AMP	956	2,452

### Feature data acquisition

#### SNP data acquisition

SNP features were extracted using Snippy (v4.6.0) (Luth et al., 2021), with variant calling performed for each sample against the reference genome GCF_000005845.2 (FASTA) and its corresponding annotation file (GFF/GTF). Core SNPs were identified with the snippy-core module, which aggregates variant calls across all isolates to generate a set of polymorphic sites that are consistently covered and callable across samples. To mitigate the influence of missing data and low-information loci, core SNPs were further filtered as follows: (i) missingness filtering, retaining sites that were callable in ≥ 95% of samples; (ii) minor allele frequency (MAF), retaining sites with MAF ≥ 1% to remove extremely rare variants; and (iii) recombination masking, in which recombinant regions were identified on the core alignment using Gubbins (v3.4.3) and subsequently masked.

#### Gene data acquisition

Each isolate genome was annotated using Bakta (v1.11.4) (Schwengers et al., 2021), which provides standardized identification and annotation of protein-coding sequences, non-coding RNAs, and other functional elements. A pangenome was then constructed with Panaroo (v1.5.2) based on the annotations from all samples. Using graph-based clustering and sequence alignment, Panaroo delineates core and accessory genes and generates a cross-sample gene presence/absence matrix, which was used to represent the retention status of each gene in each genome.

#### k-mer data acquisition

To generate reference-free sequence features, k-mer frequencies were computed for each isolate using Jellyfish (v2.3.1) (Marçais and Kingsford, 2011). To balance sequence specificity and computational efficiency, the k-mer length was set to 11 (11-mers). Canonical k-mers were used to reduce redundancy by merging each k-mer with its reverse complement. After compiling the k-mer set across all isolates, features were filtered based on cohort-wide detection rates to reduce noise and low-information variables; only k-mers present in ≥ 1% and ≤ 99% of isolates were retained for downstream analyses.

#### Feature selection

To reduce dimensionality and prioritize features strongly associated with antimicrobial resistance phenotypes, feature selection was performed strictly within the training set during each modeling procedure to avoid data leakage. For each candidate feature, the presence proportion in the resistant (R) and the susceptible (S) groups were calculated, and the absolute difference between the two proportions was used as a discriminative score (Score = | P_*R*_-P_*S*_|). Features were ranked in descending order by score, and the top 1,000 features were retained for subsequent machine-learning model training. In addition, chi-square tests and odds ratios (ORs) were calculated using the training data to identify resistance-associated features (*P*< 0.05 and OR > 2). The resulting selected features were then applied to the corresponding test set for model evaluation.

#### Model construction and performance analysis

Samples were randomly divided into training and test sets at a 60:40 ratio using the createDataPartition function in the R package caret. For each feature type (SNPs, gene and k-mers), models were trained using the H_2_O framework with 10-fold cross-validation (nfolds = 10), including deep learning (h2o.deeplearning), gradient boosting machines (h2o.gbm), generalized linear models (h2o.glm), and random forests (h2o.randomForest). Models were integrated using h2o.stackedEnsemble, and the resulting 12 models across the three data types were further combined into a final ensemble model. Final model performance was then evaluated on the held-out test set.

**FIGURE 1:**
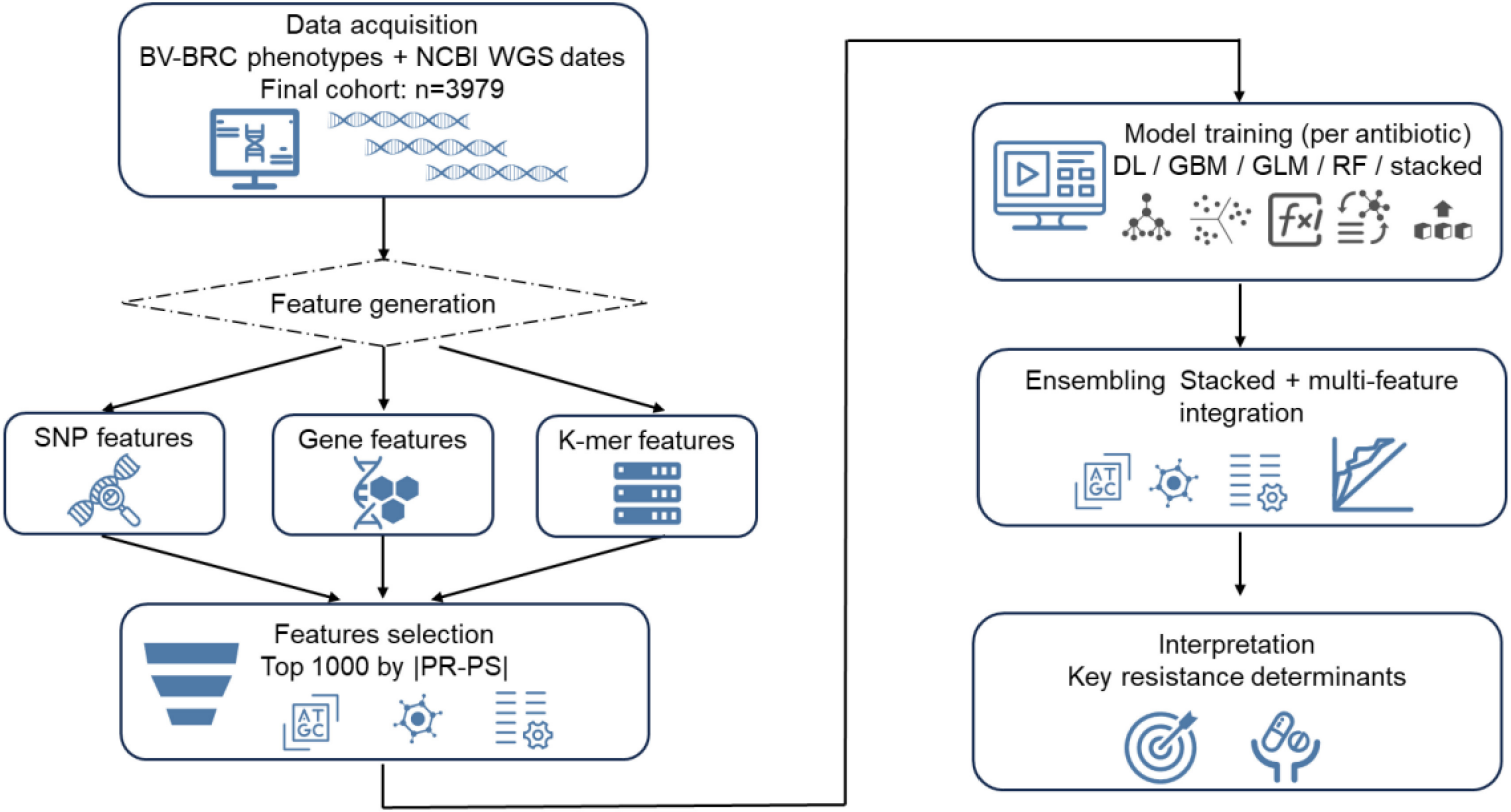
The process of machine learning classifier construction.

Receiver operating characteristic (ROC) curves were generated based on predicted probabilities from the validation set using the roc() function in the R package pROC, and the area under the ROC curve (AUC) was calculated to evaluate overall discriminative performance. The optimal probability threshold was determined by maximizing the Youden index on the ROC curve, and predicted probabilities were then converted into binary class labels to generate confusion matrices (TP, FP, TN, FN). Sensitivity, specificity, accuracy, and F1-score were then calculated to compare the performance of different classifiers in predicting resistant versus susceptible phenotypes. The workflow of this study is shown in Figure 1.

## Results

### Statistical analysis of strain origin and AST phenotypes

Whole-genome sequencing data from 1952 *Escherichia coli* isolates were analyzed in this study. Because some antibiotics had relatively few resistant or susceptible isolates, which potentially increase the risk of model overfitting and biased prediction, we restricted our analysis to 10 commonly used antibiotics. Notably, sample sizes differed substantially across antibiotics, and varying degrees of class imbalance were observed.

**FIGURE 2:**
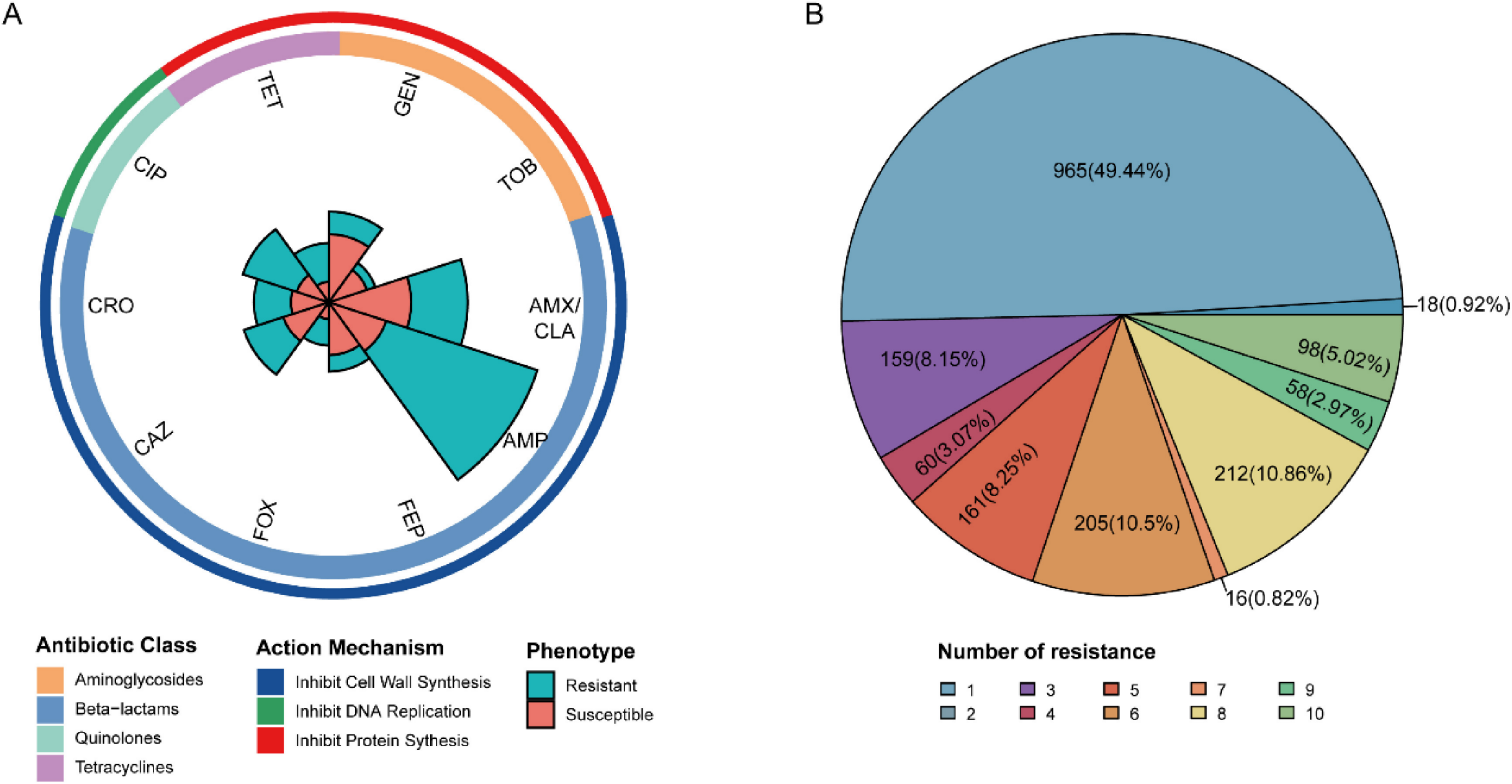
Resistance phenotypes and genotypes of publicly available genomes. **(A)** Antimicrobial susceptibility profiles for 10 antibiotics. **(B)** Distribution of resistance burden across isolates.

In terms of antibiotic classes, beta-lactams predominated in this dataset, including multiple cephalosporins and penicillins/β-lactamase inhibitor combinations. The cephalosporins comprised cefepime (FEP; *S* = 1,201, *R* = 211), cefoxitin (FOX; *S* = 765, *R* = 402), ceftriaxone (CRO; *S* = 696, *R* = 444), and ceftazidime (CAZ; *S* = 2,349, *R* = 713). The penicillins and combination agents included ampicillin (AMP; *S* = 956, *R* = 2,452) and amoxicillin/clavulanic acid (AMX/CLA; *S* = 2,070, *R* = 1,215). Additionally, aminoglycosides (gentamicin, GEN; *S* = 2,765, *R* = 580; tobramycin, TOB; *S* = 1,214, *R* = 368), a tetracycline (TET; *S* = 440, *R* = 599), and a quinolone (ciprofloxacin, CIP; *S* = 2,195, *R* = 1,034) were included (Figure 2A).

Regarding class balance, several antibiotics showed markedly resistance-skewed distributions. This imbalance was most pronounced for ampicillin (AMP), where resistant isolates (*R* = 2,452) substantially outnumbered susceptible isolates (*S* = 956), indicating a high resistance burden in this cohort. Among the 1,952 isolates, nearly half (49.44%) were resistant to two antibiotics. Notably, 98 isolates (5.02%) were resistant to all 10 antibiotics, whereas only 18 isolates were resistant to a single antibiotic (Figure 2B).

### AMR phenotype prediction performance of optimal models

**FIGURE 3:**
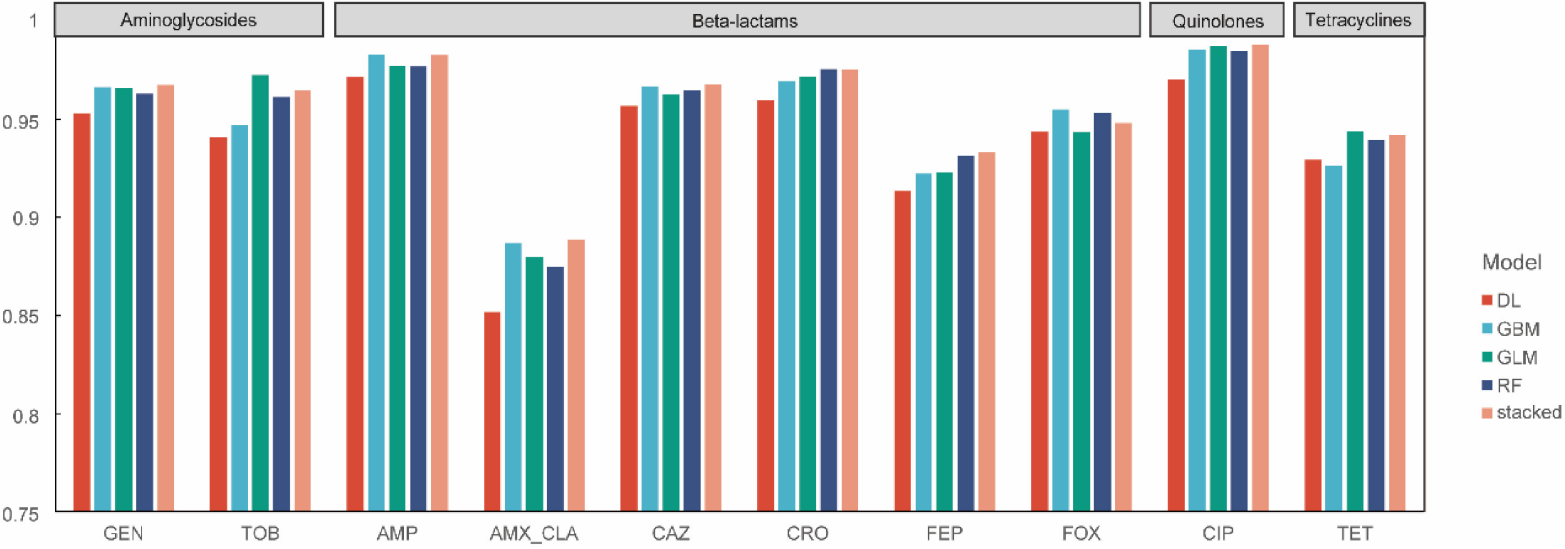
Comparison of performances between machine learning methods.

Figure 3 summarizes the AUC performance of four machine-learning algorithms (DL, GBM, GLM, and RF) and a stacked ensemble model across 10 antibiotics representing aminoglycosides, β-lactams, quinolones, and tetracyclines. Overall, all models demonstrated strong discriminatory ability for most antibiotics, with AUC values predominantly exceeding 0.90. GBM and the stacked ensemble achieved the highest or near-highest AUCs for most antibiotics, indicating that ensemble strategies provide more robust and consistent predictive performance across antibiotic-specific prediction tasks. GLM and RF exhibited comparable performance and were slightly inferior to GBM and the stacked ensemble, while still maintaining high AUC values.

When stratified by antibiotic class, models achieved near-ceiling performance for the aminoglycosides (GEN, TOB) and the fluoroquinolone (CIP), with AUCs approaching 1.0. By contrast, predictive performance was more heterogeneous among β-lactams. Notably, AMX/CLA exhibited a substantially lower AUC than the other agents. This finding suggested that resistance mechanisms associated with AMX/CLA may be more complex or less well captured by the current feature set, and may represent a key driver of the observed performance differences among antibiotics.

### Model performance evaluation

**FIGURE 4:**
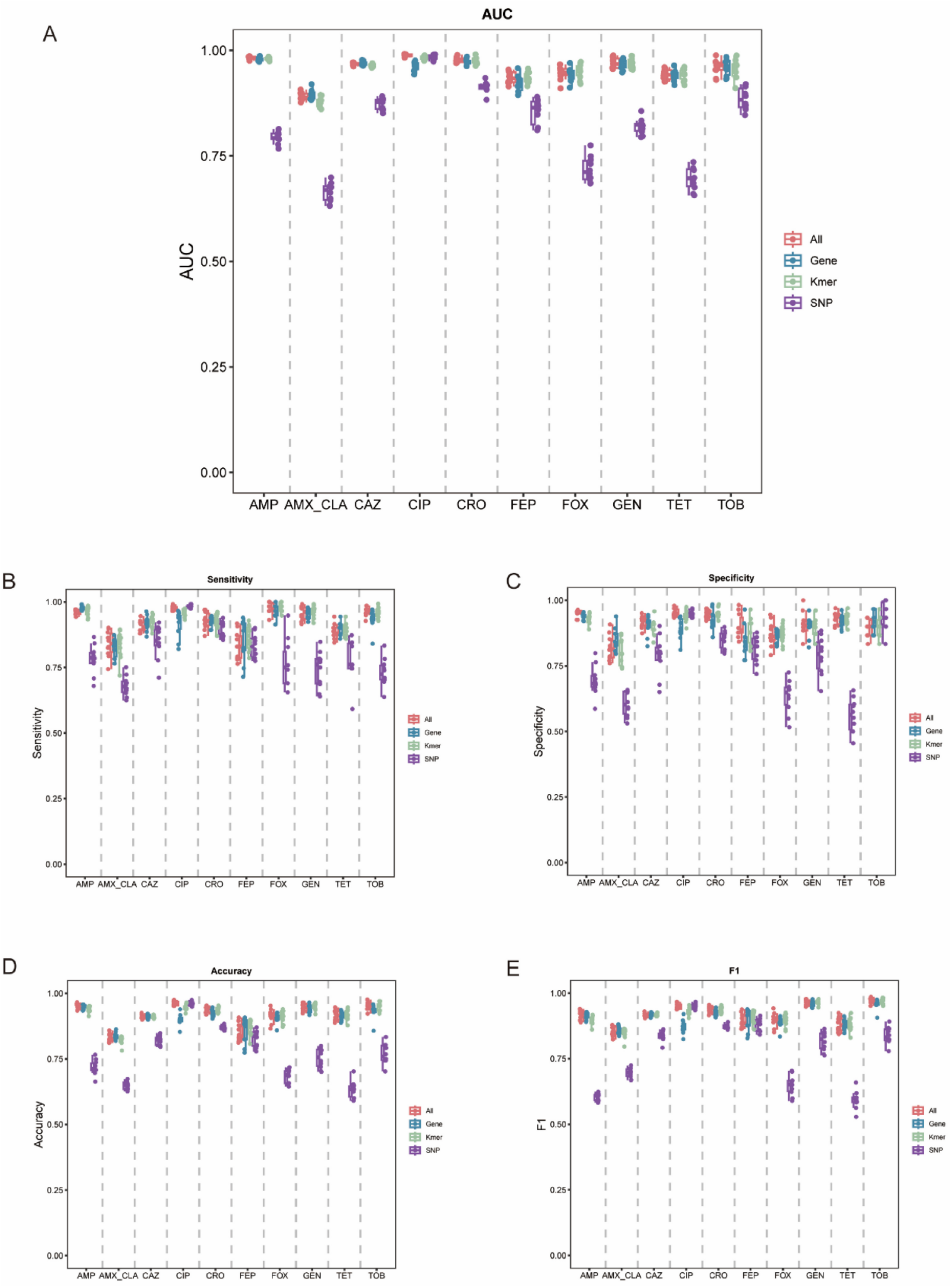
Performance of ten antibiotic resistance prediction models constructed using different feature types. **(A)** Area under the ROC curve (AUC). **(B)** Sensitivity. **(C)** Specificity. **(D)** Accuracy. **(E)** F1 score.

The performance of AMR phenotype prediction models constructed using different feature types across the 10 antibiotics were summarized in Figure 4. Overall, all models achieved effective predictive performance, with AUC values ranging from 0.6691 to 0.9879. The integrated multi-feature (“All”) model (AUC: 0.8888–0.9879) and the gene-based model (AUC: 0.8936–0.9787) showed overall higher AUCs than the SNP-based model (AUC: 0.6691–0.9841) and the k-mer-based model (AUC: 0.8801–0.9812), exhibiting a higher distribution of AUC values and greater stability (Figure 4A). These results indicated that gene-level features provide stronger discrimination between resistant and susceptible phenotypes, and that multi-feature integration can maintain or further improve overall performance. Consistent with the AUC results, the Gene and All models also generally outperformed the SNP and k-mer models in sensitivity, specificity, accuracy, and F1-score (Figures 4B–E), indicating more balanced and reliable performance in identifying both resistant and susceptible isolates.

In contrast, the SNP-based models showed lower and more variable performance across multiple antibiotics, with decreases in AUC as well as in sensitivity, specificity, accuracy, and F1-score. This decline was particularly pronounced for β-lactams (AMP, AMX/CLA, CAZ, CRO, FEP, FOX) and tetracycline (TET), indicating that SNP features alone may be insufficient to capture key resistance determinants for these agents. The k-mer-based models performed intermediate between the Gene/All and SNP models; although they achieved relatively high sensitivity and accuracy for certain antibiotics, their overall performance remained inferior to that of the gene-based and multi-feature models.

### Feature importance assessment

**FIGURE 5:**
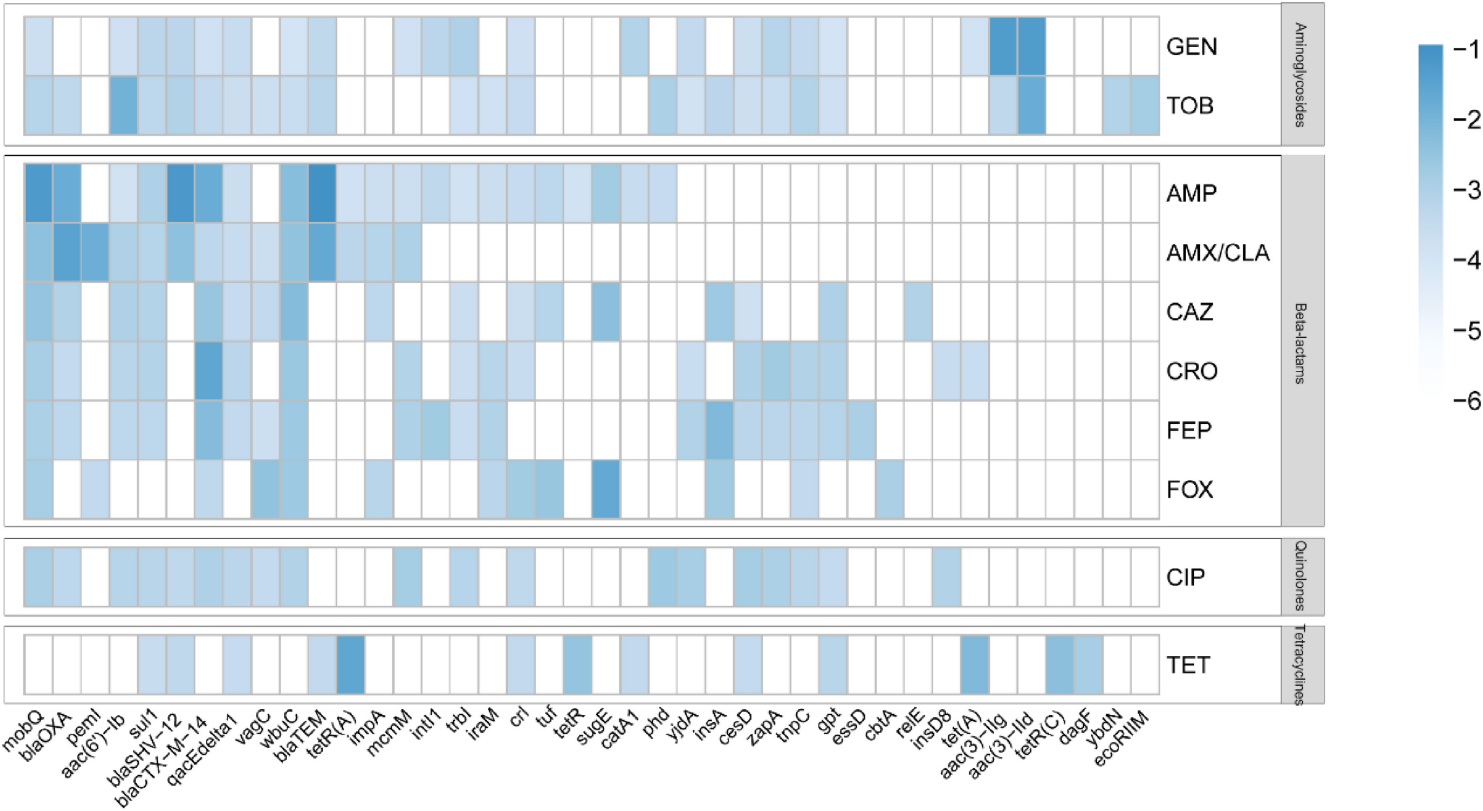
Genomic landscape of gene contributions to discriminating AMR phenotypes across ten antibiotics in *E. coli* (blue markers indicate genes with higher contributions to distinguishing resistant vs. susceptible isolates for a given antibiotic, whereas blank cells denote lower contributions).

To systematically delineate the genetic determinants underlying *Escherichia coli* resistance phenotypes across antibiotic classes, we conducted feature selection and statistical association analyses by contrasting resistant and susceptible isolates for each antibiotic. This analysis ultimately identified 40 core genes with significant contributions to the resistance prediction models and further resolved gene-specific contribution patterns across different drug resistance phenotypes (Figure 5 and Supplementary Figure 1). Notably, *mobQ*, *blaCTX-M-14*, and *wbuC* contributed significantly to the predictive models for aminoglycosides, β-lactams, and quinolones, with their strongest effects observed in the β-lactam models.

Within the aminoglycoside class (GEN and TOB), the aminoglycoside-modifying enzyme genes *aac(3)-IIg* and *aac(3)-IId* exhibited markedly enriched association signals, consistent with established mechanisms of aminoglycoside resistance. Their contributions were substantially higher in the GEN model than in the TOB model, suggesting a subtype preference in the genetic architecture driving resistance among different aminoglycosides. For Beta-lactams, multiple β-lactamase genes, including *blaTEM*, *blaSHV-12*, *blaCTX-M-14*, and *blaOXA*, exhibited strong association signals. Specifically, *blaCTX-M-14* contributed much more to resistance against third-generation cephalosporins (CAZ, CRO, and FEP) than to penicillins, whereas *blaTEM* showed a preferential contribution to penicillin resistance, highlighting the substrate specificity of distinct β-lactamases. In quinolones, key resistance-associated genes (e.g., *mobQ*, *blaOXA*, and *aac(6’)-Ib*) displayed no significant differences in contribution. For tetracyclines, *tetR(A)* displayed the highest contribution, followed by *tet(A)* and *tetR(C)*.

## Discussion

AMR has emerged as a major obstacle to precision management of infectious diseases. Conventional antimicrobial susceptibility testing (AST) is culture dependent and time-consuming, which limits its utility for timely clinical decision-making (van Belkum et al., 2020; Hattab et al., 2024). With the decreasing cost of WGS and the expanding availability of genomic datasets, ML-based genotype-to-phenotype mapping has emerged as a promising strategy for rapid prediction of antibiotic resistance (Anahtar et al., 2021; Ardila et al., 2024). In this study, we developed and systematically compared ML models for predicting resistance of *Escherichia coli* to 10 commonly used antibiotics using different feature representations (gene, SNPs, k-mers, and their integration) and multiple algorithms. Overall, the models demonstrated strong predictive performance and highlighted that the optimal combination of algorithm and feature type varies across antibiotic-specific prediction tasks.

In our dataset, the numbers of resistant and susceptible isolates differed markedly across antibiotics, with class imbalance particularly pronounced among β-lactams. Such imbalance can bias model training toward the majority class and consequently compromise clinically relevant performance metrics, even when threshold-independent measures such as the AUC appear favorable (Owusu-Adjei et al., 2023). To reduce the risk of overfitting driven by small class sizes, we limited our analyses to 10 antibiotics with relatively sufficient sample counts and assessed model performance using multiple complementary metrics. Notably, multidrug resistance was prevalent in our cohort, with nearly half of the isolates resistant to two antibiotics and a substantial subset resistant to all 10 antibiotics. This distribution suggested that resistance determinants may co-occur and disseminate in a modular manner. Although such co-occurrence may improve model learnability for certain prediction tasks (Powell-Romero et al., 2023), it can also introduce cross-antibiotic collinearity, thereby complicating discrimination for antibiotics with more complex or heterogeneous resistance mechanisms.

Across the evaluated algorithms, GBM and stacked ensemble models achieved the highest or near-highest AUCs for most antibiotics and exhibited more consistent performance across tasks. These results indicated that ensemble approaches are well suited to high-dimensional, sparse genomic data, as they can capture nonlinear effects and feature interactions while reducing the instability inherent to any single learner. In contrast, GLM and RF models generally produced slightly lower, yet still robust, AUCs, indicating that AMR phenotypes in *E. coli* are largely separable within the current feature space. Nevertheless, the relative advantage of any given algorithm is likely antibiotic dependent, reflecting both the predominant resistance mechanisms and the extent to which these mechanisms are represented by the chosen feature set (Pesesky et al., 2016; Anahtar et al., 2021). These findings highlight the value of antibiotic-specific model selection rather than adopting a one-size-fits-all strategy.

When comparing feature representations, gene-based models and the integrated (All) models consistently outperformed SNP and k-mer, showing higher AUCs and greater stability across antibiotics. This observation was biologically plausible, as resistance in *E. coli* across multiple drug classes is frequently driven by acquired resistance genes (e.g., β-lactamases), for which gene provides a strong and direct predictive signal (Hussain et al., 2021; Nasrollahian et al., 2024; Kerek et al., 2025). By contrast, SNP features are expected to be most informative for antibiotics whose resistance is primarily determined by chromosomal point mutations (Shi et al., 2019); however, when resistance is largely mediated by horizontal gene transfer, SNP signals tend to be weaker and more diffuse, and they are more susceptible to confounding from population structure, reference genome selection, and missing data (Mobegi et al., 2017; Sezmis et al., 2023). Although k-mers can capture sequence signals associated with gene acquisition, point mutations, and structural variation simultaneously, their extreme dimensionality, redundancy, and limited interpretability may impair model stability and hinder translational implementation, particularly when sample sizes are small or feature selection is insufficient (Jaillard et al., 2020).

Importantly, we observed pronounced differences in prediction difficulty across antibiotic classes. For aminoglycosides (GEN and TOB) and the ciprofloxacin (CIP), AUC values were near saturation, indicating that the relevant resistance signals are relatively concentrated and are well captured by the current feature sets. In contrast, β-lactams exhibited greater within-class heterogeneity, and AMX/CLA showed a markedly lower AUC than the other agents, suggesting that resistance to this β-lactam/β-lactamase inhibitor combination is more complex and not fully represented by our feature framework in *E. coli*. Relative to single-agent β-lactams, resistance to β-lactam/β-lactamase inhibitor combinations often reflects multiple, overlapping mechanisms, including variation in β-lactamase subtype and expression level, promoter or other regulatory changes, porin alterations, efflux pump activation, and mobile element-mediated upregulation (Yahav et al., 2020). Accordingly, gene presence/absence alone may be insufficient to comprehensively capture the determinants of resistance for these agents. However, this observation should not be directly generalized to other bacterial species, as the genetic basis and relative contribution of AMX/CLA resistance mechanisms may differ across organisms.

Feature importance analysis further demonstrated that the key genes prioritized by our models were largely concordant with well-established resistance mechanisms. For aminoglycosides, the aminoglycoside-modifying enzyme genes *aac(3)-IIg* and *aac(3)-IId* were among the most influential predictors, consistent with the central role of drug-modifying enzymes in mediating resistance to this class (Nicoloff and Andersson, 2016). For β-lactams, multiple β-lactamase genes, including *blaTEM*, *blaSHV-12*, *blaCTX-M-14*, and *blaOXA*, showed strong associations with resistance. Notably, *blaCTX-M-14* contributed more prominently to predictions for third-generation cephalosporins (CAZ, CRO, and FEP), whereas *blaTEM* was more strongly linked to penicillin resistance, reflecting known differences in β-lactamase substrate spectra (Bush and Bradford, 2016). For tetracyclines, *tetR(A)* and *tet(A)* emerged as key features, aligning with canonical efflux- and regulation-mediated resistance mechanisms (De Gaetano et al., 2023). In addition, genes such as *mobQ* contributed across multiple antibiotic classes, suggesting that they may serve as proxies for plasmid mobility or co-carriage of resistance modules rather than functioning as direct resistance determinants (Pacheco-Acosta et al., 2025). Collectively, these results indicate that ML models can provide not only accurate phenotype prediction but also a data-driven framework to prioritize candidate molecular markers and dissemination-related genomic features for downstream mechanistic studies and genomic surveillance (Xu et al., 2022).

Despite the encouraging performance, there were several limitations should be acknowledged. First, although we prioritized antibiotics with relatively sufficient sample sizes to reduce the risk of overfitting, substantial heterogeneity in resistant-to-susceptible ratios remained across drugs, which may have affected model performance and limited clinical utility. Second, model evaluation relied on repeated random split validation without independent external validation, thereby limiting assessment of generalizability across different clinical and epidemiological settings. Third, the current study focused on binary resistant/susceptible classification based on AST results and did not incorporate MIC data, which may be more informative for precision treatment decisions. Finally, we did not benchmark our framework against existing dedicated AMR prediction tools, such as PhenotypeSeeker, Kover, PanKA, or PanPred. Future studies should address these limitations through external validation, MIC-based modeling, and standardized benchmarking to improve the model’s translational relevance to real-world treatment outcomes.

Overall, this study provides a comparative evaluation of WGS-derived features and commonly used machine-learning classifiers for AMR prediction in *Escherichia coli*. Our findings highlight the relative utility of different genomic feature types across antibiotic-specific tasks and may help inform practical AMR prediction workflows. Future work should focus on antibiotics with more complex resistance determinants, incorporate a broader range of genomic and phenotypic features, and further validate model performance in independent datasets to support translation into clinical practice.

## Data Availability

The original contributions presented in the study are included in the article/Supplementary material, further inquiries can be directed to the corresponding authors.
