# Supplementary material for: Utilizing whole genome sequencing data for machine learning driven prediction of antibiotic resistance in Escherichia coli
Source: Front Microbiol. 2026 Apr 28;17:1842717. doi: 10.3389/fmicb.2026.1842717 (PMC13160890; doi:10.3389/fmicb.2026.1842717)
Supplement: Supplementary file 1 [file Table_1.docx]

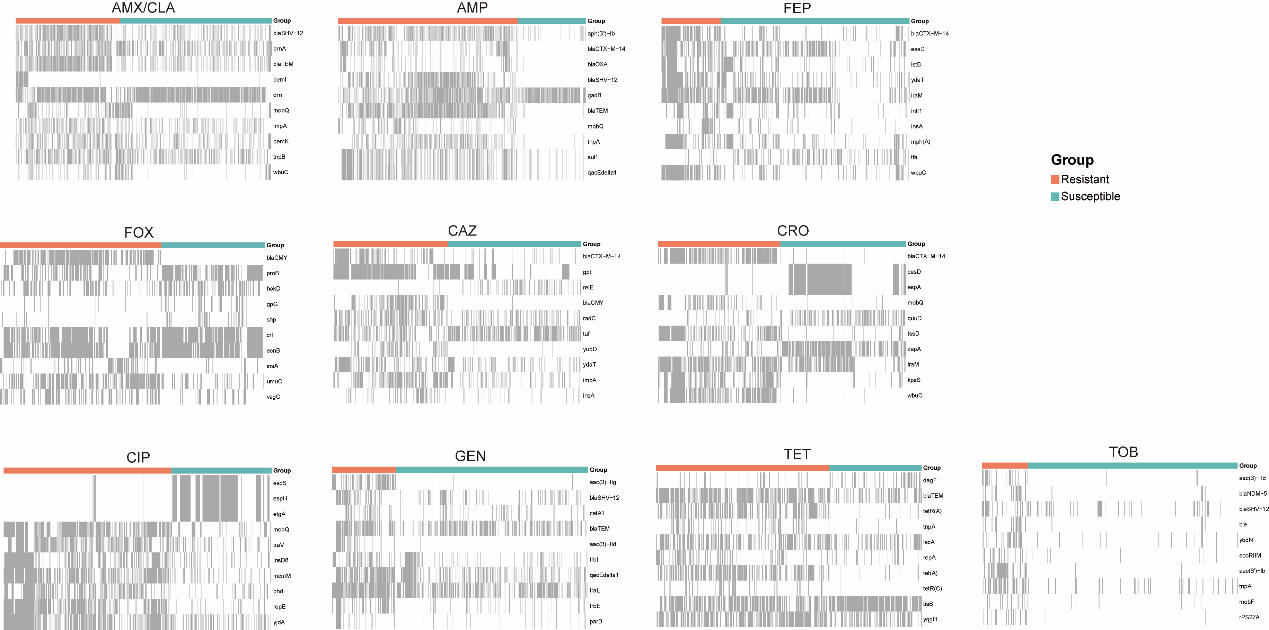


Supplementary Figure 1. Identification of informative AMR features with machine learning using the WGS-AST pipeline. Heatmap showing the most influential AMR features selected from the training sets for each antibiotic.
